# Metformin as an Enhancer for the Treatment of Chemoresistant CD34+ Acute Myeloid Leukemia Cells

**DOI:** 10.3390/genes15050648

**Published:** 2024-05-20

**Authors:** Indre Krastinaite, Sergej Charkavliuk, Ruta Navakauskiene, Veronika Viktorija Borutinskaite

**Affiliations:** Department of Molecular Cell Biology, Institute of Biochemistry, Life Sciences Center, Vilnius University, Sauletekio Av. 7, LT-10257 Vilnius, Lithuania; indre.krastinaite@gmail.com (I.K.); scharkavliuk@gmail.com (S.C.); ruta.navakauskiene@bchi.vu.lt (R.N.)

**Keywords:** leukemia stem cells, KG-1a, cell surface markers, venetoclax, metformin, S63845, cytarabine, idarubicin

## Abstract

Acute myeloid leukemia is the second most frequent type of leukemia in adults. Due to a high risk of development of chemoresistance to first-line chemotherapy, the survival rate of patients in a 5-year period is below 30%. One of the reasons is that the AML population is heterogeneous, with cell populations partly composed of very primitive CD34+CD38- hematopoietic stem/progenitor cells, which are often resistant to chemotherapy. First-line treatment with cytarabine and idarubicin fails to inhibit the proliferation of CD34+CD38- cells. In this study, we investigated Metformin’s effect with or without first-line conventional chemotherapy, or with other drugs like venetoclax and S63845, on primitive and undifferentiated CD34+ AML cells in order to explore the potential of Metformin or S63845 to serve as adjuvant therapy for AML. We found that first-line conventional chemotherapy treatment inhibited the growth of cells and arrested the cells in the S phase of the cell cycle; however, metformin affected the accumulation of cells in the G2/M phase. We observed that CD34+ KG1a cells respond better to lower doses of cytarabine or idarubicin in combination with metformin. Also, we determined that treatment with cytarabine, venetoclax, and S63845 downregulated the strong tendency of CD34+ KG1a cells to form cell aggregates in culture due to the downregulation of leukemic stem cell markers like CD34 and CD44, as well as adhesion markers. Also, we found that idarubicin slightly upregulated myeloid differentiation markers, CD11b and CD14. Treatment with cytarabine, idarubicin, venetoclax, metformin, and S63845 upregulated some cell surface markers like HLA-DR expression, and metformin upregulated CD9, CD31, and CD105 cell surface marker expression. In conclusion, we believe that metformin has the potential to be used as an adjuvant in the treatment of resistant-to-first-line-chemotherapy AML cells. Also, we believe that the results of our study will stimulate further research and the potential use of changes in the expression of cell surface markers in the development of new therapeutic strategies.

## 1. Introduction

Acute myeloid leukemia (AML) is an aggressive form of hematologic cancer of the myeloid lineage. The failure of blood cell differentiation, the buildup of immature cells (blasts) in bone marrow and/or peripheral blood, and unregulated cell division and development characterize this oncology [1]. Moreover, the clinical treatment of AML in the past 40 years has not changed and includes 7–10 days of treatment with cytarabine combined with 3 days of anthracycline treatment as remission induction therapy [2].

The unsatisfactory survival rates associated with this cancer are the result of high rates of adaptation to first-line therapy with medications such as cytarabine, daunorubicin, or idarubicin. More than 60% of elderly patients cannot be treated with standard chemotherapy due to secondary diseases and poor health, and treatment failed in over 85% of patients [1,3]. First-line therapy of eligible older patients continues to be chemotherapy with a combination of anthracycline and cytosine–arabinoside as a backbone; however, the combination of venetoclax and azacitidine or low-dose cytarabine, or low-dose cytarabine with glasdegib, could be the new standard of comparison for persons unfit for intensive therapy. Moreover, in selected studies, the survival of older patients with AML did not exceed 50% in 1 year, and primary resistance to venetoclax was frequent, remaining an unmet clinical need [4,5,6]. To improve venetoclax treatment outcomes, it was shown that a combination of venetoclax with other agents such as S63845, an MCL1 inhibitor, reduced the number of leukemic cells, indicating that MCL1 (S63845) and BCL-2 (venetoclax) inhibitors are synergistic and lead to T-ALL cell apoptosis [7].

Chemoresistance in AML, where cancer cells become less responsive or resistant to chemotherapy, is a complex phenomenon with various contributing factors [8]. AML is a heterogeneous disease, and over time, cancer cells can undergo clonal evolution, leading to the emergence of subpopulations of cells with different genetic profiles. Some of these subpopulations like cancer stem cells can self-renew, differentiate into various cell types, and may be more resistant to chemotherapy, contributing to treatment failure and relapse. It was shown that this leukemic stem cell (LSC) population is CD34+CD38- and has a strong ability to reinitiate the same leukemia in mice, whereas CD34+CD38+ cells do not have such an ability [9]. As previously demonstrated for many types of stem cells, leukemic stem cells have a low level of reactive oxygen species (ROS-low) and reside in a unique state of mitochondrial dynamics [10]. Studies on the basic functions of cellular metabolic reprogramming in tumor formation and progression have recently received increased attention. Metformin, a widely used oral medication for the treatment of type 2 diabetes, has garnered interest in cancer research due to its potential anti-cancer properties, including its ability to target cancer stem cells [11]. It is known that different adhesion molecules play crucial roles in the regulation of LSC proliferation through their interactions with the bone marrow microenvironment. Also, metformin has been shown to downregulate the expression of stem cell markers such as CD44 and ALDH in cancer cells, potentially impacting LSC properties [12]. Adhesion molecules and their downstream signaling pathways can activate stemness-related transcription factors and signaling pathways in LSCs, promoting their self-renewal and proliferation. Also, adhesion to the bone marrow niche can protect LSCs from apoptosis induced by chemotherapy or other stressors. It was shown that LSCs require interaction with a niche to maintain their stem cell properties, and new strategies to disrupt that interaction can lead to novel therapeutic strategies to eliminate quiescent AML LSCs [13].

Due to resistance to first-line chemotherapy, new therapeutic techniques for relapsed/refractory AML must be developed [14,15]. As new, innovative alternatives are being developed, repurposing old drugs for new applications is a potential method for relieving and improving the conditions of people suffering from serious illnesses [15].

The purpose of this study is to look at the anti-leukemic effects of metformin (approved for diabetes) alone or in combination with first-line chemotherapy or other drugs like venetoclax (BCL2 inhibitor, approved for chronic lymphocytic leukemia (CLL), AML) and S63845 (MCL1 inhibitor, experimental) on the primitive and undifferentiated CD34+ AML cell line to improve AML treatment efficacy.

## 2. Materials and Methods

### 2.1. Human Acute Myeloid Leukemia Cell Lines and Their Cultivation

The CD34+ AML cell line (KG1a) was used in this research. KG-1a cells are a subtype of the progenitor cell line—KG-1. Various abnormalities developed in the KG-1 cells after 35 rounds of culture. These anomalies are mostly dependent on the karyotype, namely chromosomal rearrangements. For example, the first chromosome received a doubled long arm, the sixteenth chromosome’s deleted short arm, two copies of the twenty-second chromosome, and a lack of the Y chromosome, among other things [16]. The KG-1a cells (ATCC, Manassas, VA, USA) were stored, maintained, and cultivated according to “Handling information” (atcc.org, KG-1a–CCL-246.1). Cultivation was performed using IMDM media with 20% of Fetal Bovine Serum and 1% of Penicillin/Streptomycin (Gibco, Carlsbad, CA, USA) in the BINDER incubator (BINDER GmbH, Tuttlingen, Germany).

### 2.2. Cell Viability and Proliferation Analysis

The starting cell seeding density was 0.5–0.7 × 10^6^ viable cells/mL. The cell line was incubated with cytarabine (Cayman Chemical Company, Ann Arbor, MI, USA)**,** idarubicin (MilliporeSigma, Burlington, MA, USA), metformin (Cayman Chemical Company, Ann Arbor, MI, USA), venetoclax (Cayman Chemical Company, Ann Arbor, MI, USA), and S63845 (Cayman Chemical Company, Ann Arbor, MI, USA)—alone or in different combinations for the duration of 96 h.

During the 96 h of treatment period, cell growth characteristics were monitored using trypan blue staining. Cell suspension was mixed in equal portions with 0.2% Trypan blue (MilliporeSigma, Burlington, MA, USA). The cells were then counted in a hemocytometer (Neubauer-improved, 0.1 mm depth of the chamber; Paul Marienfeld GmbH & Co. KG, Lau-da-Königshofen, Germany).

The viability of the cell cultures was estimated using 7AAD staining (Miltenyi Biotec, Inc., Auburn, CA, USA) following the manufacturer’s instructions.

Antiproliferative IC50 values and metabolic activity were evaluated using the XTT Cell Proliferation Assay Kit (ATTC, Manassas, VA, USA) following the manufacturer’s instructions.

### 2.3. Cell Cycle Analysis

After 72 h, the 1 × 10^6^ cells were centrifuged at 4 °C at 300× *g* for 5 min in an Eppendorf 5810 R centrifuge, and the cell growth medium was discarded. The cells were washed twice with 1X PBS before the 1X PBS was discarded. All groups were fixed overnight at −20 °C by progressively adding 3.3 mL of ice-cold 96% ethanol. The cells were centrifuged at 4 °C at 500× *g* for 10 min the next day, and the ethanol was discarded. After being washed twice with 1X PBS, the cells were suspended in 500 µL of ice-cold PBS. RNAse A (50 µg/mL) (ThermoFisher Scientific, Waltham, MA, USA) was used to remove RNA, then propidium iodide (25 µL) was added to the cell solution and incubated at room temperature for 30 min. The cells were examined using the Guava^®^ easyCyteTM Flow Cytometer from Luminex Corporation (Luminex Corporation, Austin, TX, USA). Guavasoft 3.3 software was used to calculate the proportion of cells in G0/G1, S, and G2/M phases.

### 2.4. RNA Extraction and Purification

The growth medium was removed after centrifuging all groups at 300× *g* for 5 min with an Eppendorf 5810 R centrifuge (Eppendorf, Hamburg, Germany). Pipetting was used to combine 800 µL of Tri reagent (Zymo Research, Irvine, CA, USA) into media-free cells, which were then incubated for 5 min at room temperature. Following the incubation, 213 µL of chloroform (Millipore Sigma, Burlington, MA, USA) was added to the lysate mixture, vortexed for 15 s to mix, then incubated at room temperature for 10 min. The top phase of the chloroform/lysate solution was transferred to freshly labeled sterile 1.5 mL tubes after centrifugation at 12,000× *g* for 15 min at 4 °C with an Eppendorf 5417 R centrifuge (Eppendorf, Hamburg, Germany). The RNA was condensed in 533 µL of isopropanol (MilliporeSigma, Burlington, MA, USA), vortexed, and incubated at room temperature for 10 min. The samples were centrifuged for 10 min at 12,000× *g* at 4 °C after incubation, and the isopropanol was discarded. The extracted total RNA was washed with 75% ice-cold ethanol (Vilniaus degtinė, Vilnius, Lithuania) and centrifuged for 5 min at 4 °C at 7500× *g*. Following that, the ethanol was removed. The remaining ethanol was allowed to dry for 5 min at ambient temperature before being suspended in 20 µL of DEPC water.

Before proceeding, the concentration of total RNA was determined using a NanoPhotometer (Implen GmbH, München, Germany) at λ = 260, λ = 230, λ and = 280 nm

### 2.5. cDNA Synthesis and Real-Time Polymerase Chain Reaction

The RNA was converted to cDNA using the LunaScript^®^ RT SuperMix Kit (ID: #E3010, New England Biolabs, Ipswich, MA, USA) and SensiFAST^TM^ cDNA Synthesis Kit (ID: BIO-65054, Meridian Bioscience Inc., Cincinnati, OH, USA) with Arktik Thermal Cycler (ThermoFisher Scientific, Waltham, MA, USA) according to manufacturer recommendations and protocols. After that, the cDNA was diluted to a final cDNA concentration of 1.25 ng and stored at −20 °C.

The quantitative polymerase chain reaction (qPCR) was carried out on QIAGEN’s Rotor-Gene Q real-time PCR system (Qiagen, Hilden, Germany) using the Luna^®^ Universal qPCR Master Mix kit (ID: M3003S, New England Biolabs, Ipswich, MA, USA) and the SensiFAST^TM^ SYBR^®^ No-ROX Kit (ID: BIO-98005, Meridian Bioscience Inc., Cincinnati, OH, USA) according to manufacturer recommendations and protocols. The primers used in this investigation for *GAPDH* (Glyceraldehyde-3-Phosphate Dehydrogenase) were F-5′-AGTCCCTGCCACACTCAG-3′ and R-5′-TACTTTATTGATGGTACATGACAAGG-3′; for *BAX* (BCL2 Associated X, Apoptosis Regulator), they were F-5′-TGCCTCAGGATGCGTCCACCAA-3′, and R-5′-CCCCAGTTGAAGTTGCCGTCAG-3′; for *BAK1* (BCL2 Antagonist/Killer 1), they were F- 5′-TCATCGGGGACGACATCAAC-3′ and R-5′-CAAACAGGCTGGTGGCAATC-3′; for *APAF1* (Apoptotic Peptidase Activating Factor 1), they were F-5′-GGCTGTGGGAAGTCTGTATTAGC-3′ and R-5′-ACTCTCATCCTGATCCAACCG-3′; for *DAPK1* (Death Associated Protein Kinase 1), they were F-5′-GGCTGTGGGAAGTCTGTATTAGC-3′ and R-5′-ACTCTCATCCTGATCCAACCG-3′; for *VIM* (Vimentin), they were F-5′-TCTCTGAGGCTGCCAACCG-3′ and R-5′-CGAAGGTGACGAGCCATTTCC--3′; for *NCAM1* (Neural Cell Adhesion Molecule 1), they were F-5′-TGTCCGATTCATAGTCCTGTCC-3′ and R-5′-CTCACAGCGATAAGTGCCCTC-3′; and for *NCAM2* (Neural Cell Adhesion Molecule 2), they were F-5′-AGGAAGGTGTTAGGTCACGGT-3′ and R-5′-CCTTTGGCATCTGTTGCTTGA-3′.

### 2.6. Flow Cytometry Analysis

For flow cytometry analysis, KG1a cells were washed twice in 1X PBS with 1% bovine serum albumin (BSA, Sigma-Aldrich, St. Louis, MO, USA) and centrifuged at 500× *g* for 5 min +4 °C. The cells were resuspended in a 50 μL PBS with 1% BSA solution, and specific antibodies were added to 0.05 × 10^6^ cells and incubated for 30 min in the dark at +4 °C. These were the following: FITC-conjugated Anti-Hu CD9 (Cat.Nr.1F-208-T100), Anti-Hu CD11b (Cat.Nr.1F-211-T100) Anti-Hu CD73 (Cat.Nr.1F-675-T100), Anti-Hu CD10 (Cat.Nr.1F-209-T100), Anti-Hu CD44 (Cat.Nr.1F-221-T100), Anti-Hu CD47 (Cat.Nr.1F-225-T100), and Anti-Hu CD29 (Cat.Nr.1F-219-T100) from Exbio (Vestec, Czech Republic); Anti-CD34 (Cat.Nr.343604), Anti-CD14 (Cat.Nr.367129), Anti-CD31 (Cat.Nr.303110), Anti-HLA-ABC (Cat.Nr.311413), and Anti-HLA-DR (Cat.Nr.307620) from Biolegend (San Diego, CA, USA); PE-conjugated Anti-Hu CD15 (Cat.Nr.1P-213-T100), Anti-Hu CD19 (Cat.Nr.1P-305-T100), and Anti-Hu CD3 (Cat.Nr.1P-815-T100) from Exbio (Vestec, Czech Republic); anti-CD166 (Cat.Nr.343903, Biolegend, San Diego, CA, USA); and APC-conjugated Anti-Hu-SSEA4 (Cat.Nr.330419), Anti-Hu CD13 (Cat.Nr.1A-396-T100), Anti-Hu CD4 (Cat.Nr.1A-359-T100), Anti-Hu CD90 (Cat.Nr.1A-652-T100), Anti-Hu CD117 (Cat.Nr.1A-586-T100), Anti-Hu CD105 (Cat.Nr.1A-298-T100), and Anti-Hu CD8 (Cat.Nr.1A-207-T100) from Exbio (Vestec, Czech Republic). In total, 488 conjugated anti-Vimentin (Cat.Nr.9854, Cell Signaling Technology, Danvers, MA, USA), 647 conjugated anti-CD133 (Cat.Nr.53276, Cell Signaling Technology, Danvers, MA, USA), CD5 (Cat.Nr.60082, Biolegend), and CD7 (Cat.Nr.343102, Biolegend) were utilized. Isotype controls were used to calculate the percentage of positive cells in the population (%), following the manufacturer’s recommended protocol.

Cells were washed twice in PBS + 1% BSA and ultimately analyzed using a Guava^®^easyCyte™ 8HT flow cytometer (Millipore, Burlington, MA, USA) with GuavaSoft 3.3 software.

### 2.7. Statistical Analysis

The data are given in the form of mean and ± standard deviation of the mean (SEM). To determine statistical significance, a one-way ANOVA with a Tukey–Kramer test was performed.

## 3. Results

In this study, we aimed to test the antiproliferative effect of an antidiabetic drug and oxidative phosphorylation inhibitor, metformin, in combination with first-line chemotherapy agents (cytarabine, idarubicin) or with venetoclax, a BCL2 inhibitor, and S63845—an experimental MCL1 inhibitor—on the primitive and undifferentiated CD34+ AML cell line, KG1a. The cell growth, viability, cell cycle distribution, ROS production, and expression of pro-apoptotic or pro-inflammatory genes were studied. Also, we characterized the cell surface markers of untreated KG1a and their changes after treatments. The changes in genes involved in cell adhesion were also investigated in this study.

### 3.1. The Effects of Venetoclax, S63845, and Metformin Alone or Together with Conventional Treatment on Cell Growth Inhibition, Apoptosis, Viability and Cell Cycle in Chemotherapy-Resistant CD34+ KG1a Cells

**Figure 1:**
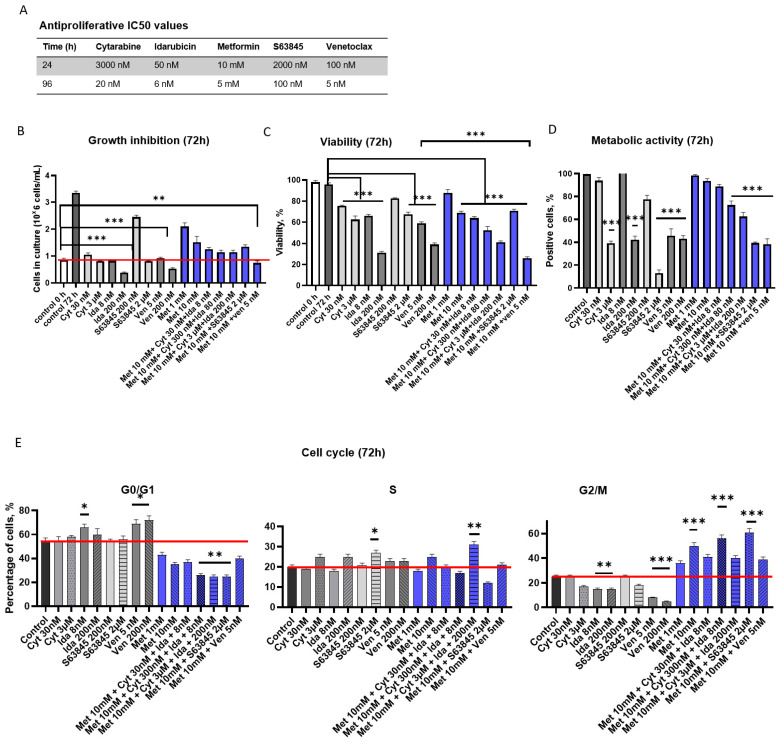
Effect of first-line chemotherapy agents—cytarabine, idarubicin, metformin, venetoclax, and S63845—alone or in combination on CD34+ AML cell line (KG1a) growth, viability, and cell cycle. (**A**) Antiproliferative IC50 values were evaluated using the XTT Cell Proliferation Assay Kit (ATTC). (**B**) Cell growth was evaluated by counting in a hemocytometer. Results are mean ± S.D. (n ≥ 3). (**C**) KG-1a cells were treated with different agents, and viability was calculated based on the % of viable (PI-) /apoptotic cells (PI) in culture by quantifying cellular DNA content after labeling with propidium iodide by flow cytometry. Results are mean ± SEM (n = 3). (**D**) The metabolic activity at 72 h was evaluated using the XTT Cell Proliferation Assay Kit (ATTC). (**E**) The distribution of KG1a cells in the G0/G1, S, and G2/M phases was detected by flow cytometry. Average ± S.D. is presented (n ≥ 3). Note: * denotes a significant difference between treated vs. control cells with *p* < 0.05, ** denotes a significant difference with *p* < 0.01, and *** denotes a significant difference with *p* < 0.005, as evaluated using one-way ANOVA with Dunnett’s post hoc test. Cyt—cytarabine; Ida—idarubicin; Ven—venetoclax; Met—metformin.

AML cells exhibit unlimited proliferation, similar to other types of tumors. An approach to develop chemotherapy drugs involves the inhibition of tumor cell proliferation. In this study, venetoclax (5 nM–20 μM), metformin (10 μM–40 mM), S63845 (200 nM–8 μM), cytarabine (30 nM–300 μm), and idarubicin (8 nM–8 μM) were used alone or in combination with metformin, and cell growth inhibition, cell cycle distribution, and apoptosis of the CD34+ KG1a cell line were assessed (Figure 1, Supplement S1).

First, XTT assays were performed to identify the IC50 values of cytarabine, idarubicin, metformin, venetoclax, and S63845 for 24–96 h in the chemotherapy-resistant KG1a cell line. The IC50 values of cytarabine for 24 h and 96 h were 3000 nM and 20 nM in KG1a cells, the IC50 values of idarubicin were 50 nM and 6 nM, the IC50 values of metformin were 10 mM and 5 mM, the IC50 values of S63845 were 2000 nM and 100 nM, and the IC50 values of venetoclax were 100 nM and 5 nM for 24 and 96 h, respectively (Figure 1A). IC50 analysis results verified that KG1a cells were resistant to cytarabine treatment during 24–72 h of treatment; only after 96 h of treatment with lower cytarabine concentration could we see the increase in apoptotic cells.

Cell growth was evaluated by a trypan blue exclusion test every day for 3 days. We revealed that cytarabine, idarubicin, and venetoclax alone inhibited cell growth after 72 h (Figure 1B). Growth inhibition occurred in a dose- and time-dependent manner (Figure 1B). The total cell number after cytarabine treatment for 72 h was 1 × 10^6^ cells per mL (Cyt 30 nM) and 0.8 × 10^6^ cells per mL (Cyt 3 μM); after idarubicin treatment, it was 0.8 nM × 10^6^ cells per mL (Ida 8 nM) and 0.38 × 10^6^ cells per mL (Ida 200 nM); after S63845 treatment, it was 2.4 × 10^6^ cells per mL (S63845 200 nM) and 0.8 × 10^6^ cells per mL (S63845 2 μM); after venetoclax treatment, it was 0.9 (Ven 5 nM) and 0.5 (Ven 200 nM); and after metformin treatment, it was 2.1 × 10^6^ cells per mL (Met 1 mM) and 1.5 × 10^6^ (Met 10 mM) (Figure 1B). The combination of metformin with cytarabine and idarubicin also inhibited proliferation of KG1a cells—1.2 (Met 10 mM + Cyt 30 nM + Ida 8 nM)), 1.1 (after Met 10 mM + Cyt 300 nM + Ida 80 nM and Met 10 mM + Cyt 3 μM + Ida 200 nM), 1.3 (Met 10 mM + S63845 2 μM), and 0.7 × 10^6^ cells per mL (Met 10 mM + Ven 5 nM).

**Figure 2:**
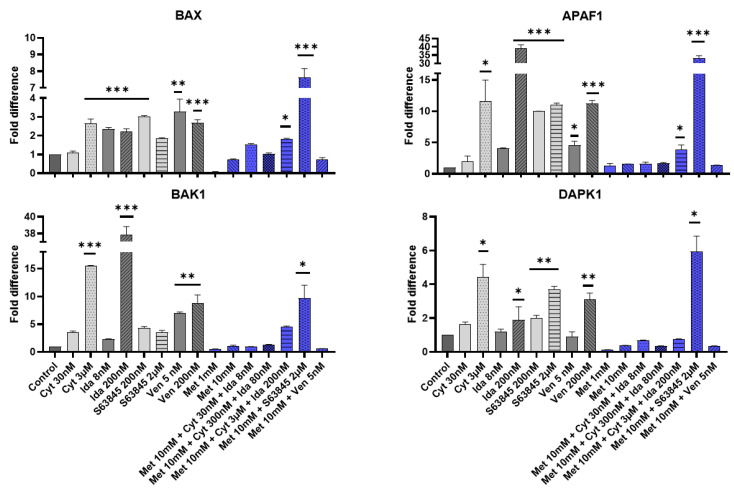
The expression changes in pro-apoptotic *BAK1*, *BAX*, *DAPK1*, and *APAF1* genes in the CD34+ AML cell line (KG1a). KG1a cells were treated with cytarabine, idarubicin, S63845, metformin, venetoclax, and combinations of these for 72 h. The qPCR results were normalized to GAPDH gene expression, and the relative gene expression was calculated with the ΔΔCt method. Note: * denotes a significant difference between treated vs. control cells with *p* < 0.05, ** denotes significant difference with *p* < 0.01, and *** denotes significant difference with *p* < 0.005. Cyt—cytarabine; Ida—idarubicin; Ven—venetoclax; Met—metformin.

Inducing apoptosis in cancer cells is a hallmark of chemotherapy medicines. In our current investigation, we employed flow cytometry to identify the impact of cytarabine, idarubicin, metformin, venetoclax, and S63845 in promoting apoptosis in CD34+ KG1a cells. To evaluate viability and cytotoxicity after 72 h, the viable (PI-) and apoptotic (PI+) cells were measured by quantifying cellular DNA content after labeling with 7AAD via flow cytometry (Figure 1C). Idarubicin (200 nM) had the most pronounced effect on the viability of KG1a cells in comparison to higher concentrations of cytarabine, venetoclax, or S63845 (Figure 1C). However, if we compare the anti-proliferative effect of lower concentration agents, we can see that idarubicin (8 nM) and venetoclax (5 nM) had similar effects and reduced viability up to 60–66%. We revealed that metformin enhanced the anti-proliferative effect of venetoclax, and after 72 h of treatment (Met + ven), the viability was 2 times lower than after 5 nM treatment with venetoclax alone (Figure 1C). Metformin and S63845 alone at lower doses showed little to no effect on KG-1a cell viability. When compared to control cells, 1 mM metformin alone did not increase the number of apoptotic cells (Figure 1C). However, 10 mM metformin increased the number of apoptotic cells up to 30% of the total population (Figure 1B and Figure 2). The most significant effect on the KG-1a cell line was observed after treatment with higher doses of idarubicin alone (70% apoptotic cells), venetoclax alone (60% apoptotic cells), and after the combination of metformin with venetoclax (78% apoptotic cells).

Metabolic activity can influence the proliferation, differentiation, and apoptosis of cells. The metabolic activity of the cells after different treatments was evaluated using the XTT Cell Proliferation Assay Kit. We detected correlation between cell growth and metabolic activity (Figure 1B,D). Metabolic activity inhibition occurred in a dose- and time-dependent manner (Figure 1D). Venetoclax (5 and 200 nM), 3 μM of cytarabine, and 200 nM of idarubicin inhibited metabolic activity 2× compared with control cells (Figure 1D). Metformin alone did not affect metabolic activity; however, in combination with venetoclax or S63845, it inhibited activity up to 2 times compared with metformin alone.

Then, the distribution of the cells in different cell cycle phases was determined by flow cytometry (Figure 1E). A 3 μM cytarabine treatment increased cell accumulation in the S phase of the cell cycle by up to 23.2% while lowering accumulation in the G2/M to 17.7%. A lower concentration of cytarabine did not affect the cell cycle (Figure 1E). Similar results were achieved after treatment with idarubicin. Treatment with venetoclax alone resulted in the greatest percentages of cell accumulation in the G0/G1 cycle phase. The increase in the G0/G1 phase was offset by a decrease in the G2/M phase. However, metformin alone or in different combinations boosted the accumulation of cells in the G2/M phase (Figure 1E). The increase in the G2/M1 phase was offset by a decrease in the G0/G1 phase.

In conclusion, the most promising outcomes on growth inhibition were seen after 72 h of treatment with cytarabine, idarubicin, venetoclax, and S63845 alone. Metformin alone, or in combination with cytarabine, idarubicin, venetoclax, or S63845, did not have such a strong effect on cell growth. However, the viability and metabolic activity of the CD34+ KG1a cell line were affected. Only metformin alone, but not in combination with other treatments, induced total ROS formation. Eventually, cytarabine, idarubicin, venetoclax, and S63845 treatment enhanced the gene expression of pro-apoptotic genes in CD34+ KG1a cells and induced cell arrest in the G0/G1 phase.

### 3.2. Expression of Pro-Apoptotic Genes after Treatment with Venetoclax, Metformin, and S63845 Alone or in Combination with Conventional Treatment on Chemotherapy-Resistant CD34+ KG1a Cells

It is known that BAK1, BAX, DAPK1, and APAF1 proteins are all key players in the intricate process of apoptosis [17]. In this study, the expression levels of pro-apoptotic *BAK1*, *BAX*, *DAPK1*, and *APAF1* genes were examined after treatments with cytarabine, idarubicin, metformin, venetoclax, and S63845 in KG-1a cells (Figure 2).

After 72 h, the *BAX* gene was increased in 3 μM cytarabine-treated cells (2.6-fold difference), idarubicin (~2.3-fold difference), and S63845-treated cells (3-fold difference). The usual combination and S63845-treated cells knocked down the *BAX* gene, and no gene expression was identified. Metformin alone or in combination with cytarabine/idarubicin had little effect on *BAX* gene expression. However, metformin in combination with S63845 boosted the upregulation of the *BAX* gene up to 7 times (Figure 2).

After 72 h of incubation, the *BAK1* gene was upregulated after 3 μM of cytarabine, 200 nM of idarubicin, and venetoclax treatment (15-, 37- and 8-fold difference, respectively). Metformin alone did not affect *BAK1* gene expression, but in combination with 200 nM of S63845, it boosted gene expression up to a 9-fold difference in comparison with S63845 alone (3,5-fold difference).

The *APAF1* gene was upregulated in the following groups: 3 μM cytarabine-treated cells (11-fold difference), 200 nM idarubicin-treated cells (39-fold difference), S63845-treated cells (10-fold difference), and venetoclax-treated cells (11-fold difference). Metformin alone did not affect *APAF1* gene expression, but in combination with 200 nM of S63845, it boosted gene expression up to a 33-fold difference in comparison with S63845 alone (10-fold difference).

After 72 h of incubation, the DAPK1 gene was upregulated after treatment with 3 μM of cytarabine (4.0-fold difference), 200 nM of idarubicin (2.0-fold difference), 2 μM of S63845 (3,7-fold difference), and 200 nM of venetoclax (3.0-fold difference) (Figure 2). Metformin alone did not affect *DAPK1* gene expression, but in combination with 200 nM of S63845, it boosted gene expression up to a 6-fold difference in comparison with 200 nM S63845 alone (2-fold difference).

### 3.3. Morphological Observations and Cell Surface Marker Expression of the CD34+ KG1a Cell Line

**Figure 3:**
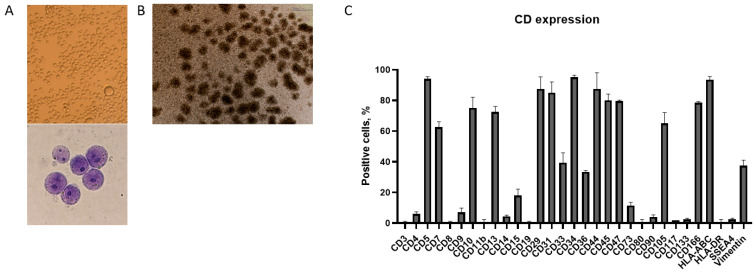
Surface phenotype analysis of the CD34+ AML cell line (KG1a) with flow cytometry. (**A**,**B**) The morphology of control cells was determined after Wright–Giemsa staining for light microscopic examination—original magnification ×20 (upper) and ×100. (**C**) The cells were stained with different surface antigens and analyzed with flow cytometry. Data are shown for each individual positive cell marker. The results are presented as mean ± S.D.

One notable characteristic of KG-1a cells is their ability to form cell aggregates or clusters [18]. In this study, we observed typical cell characteristics under a microscope; cells are generally small- to medium-sized with a round to oval shape, and also have a high nuclear-to-cytoplasmic ratio with more prominent nucleoli (Figure 3A). After 24 h of incubation, KG1a cells form aggregates (Figure 3B).

To further investigate the surface marker expression of human leukemia cell lines, KG1a, we performed flow cytometric analysis. The percentages of positive cells for each marker are shown in Figure 3C. The cells were negative for mature and differentiated hematopoietic cell markers like CD3, CD4, CD8, CD11b, CD14, and CD15 (Figure 3C). In this study, we found that KG1a cells were positive for T/B cell markers such as CD5 (up to 80%), CD7 (up to 60%), and CD10 (>60%) but negative for B-cell precursors (e.g., CD 19). We detected CD13 (70%), as CD13 expression has been associated with chemotherapy resistance in AML. KG1a cells were positive for leukemic stem cell markers such as CD34+ (95%), CD44 (80%), and CD47 (80%). We detected expressions of cell adhesion/migration/angiogenesis markers such as CD29 (85%), CD31 (80%), CD45 (80%), CD105 (65%), and CD166 (75%). KG1a cells were positive for CD33 (40%), a diagnostic AML marker. KG1a cells were HLA-ABC-positive and HLA-DR-negative (Figure 3B); vimentin was also expressed on the KG1a cell surface (40% positive).

### 3.4. S63845 Alone Can Induce KG1a Cell Differentiation; Cytarabine, Idarubicin, Metformin, and Venetoclax Can Reduce Leukemic Stem Cell Markers CD34 and CD44

**Figure 4:**
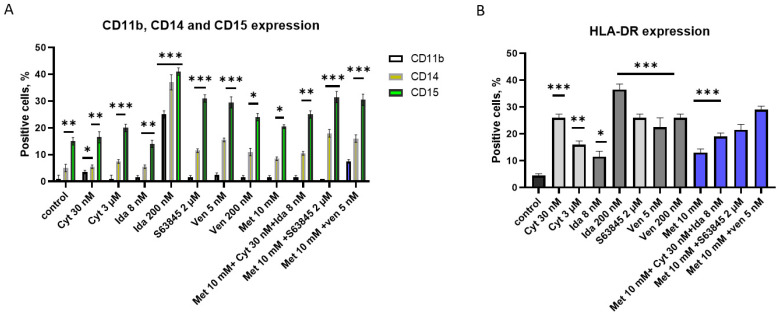
The expression of myeloid differentiation in CD11b, CD14, and CD15 markers, as well as HLA-DR markers, on KG1a and CD34+ AML cell lines after treatments. (**A**) CD11b, CD14, CD15, and (**B**) HLA-DR expression. Results are presented as mean ± S.D. Note: * denotes a significant difference between treated vs. control cells with *p* < 0.05, ** denotes a significant difference with *p* < 0.01, and *** denotes a significant difference with *p* < 0.005. Cyt—cytarabine; Ida—idarubicin; Ven—venetoclax; Met—metformin.

It is known that KG1a is in the premature stage and resistant to phorbol-diester-induced macrophage differentiation [19]. Here we studied the changes in differentiation markers CD11b, CD14, and CD15 after different treatments (Figure 4A). It is known that CD11b and CD14 are monocyte/macrophage differentiation markers, and CD15 is commonly used as a marker for granulocytic differentiation. We found that only S63845 upregulated CD11b, CD14, and CD15 expression up to 23–40%. Other treatments did not have any effect on CD11b expression in KG1a cells. We found around 15% of CD15 on the KG1a cell surface, and CD15 was upregulated very slightly after 30 nM and 3 μM of cytarabine or 8 nM of idarubicin. Other treatments upregulated CD15 up to 20–40% (Figure 4A).

Major histocompatibility complex (MHC) class II molecule HLA-DR expression on AML cells has been investigated as a prognostic marker in clinical studies. High levels of HLA-DR expression have been associated with favorable outcomes in some studies, suggesting its potential role in immune recognition and response against leukemia. Conversely, a loss or downregulation of HLA-DR expression may be associated with immune escape and disease progression [20]. In this study, we detected no HLA-DR expression in control cells (Figure 3C and Figure 4B). However, due to different treatments used in this study, we detected upregulation of HLA-DR up to 40% (Figure 4B). The largest (40%) effect was observed after treatment with 200 nM of idarubicin, and this correlated with the ability to express CD11b and CD14 markers (Figure 4A,B).

**Figure 5:**
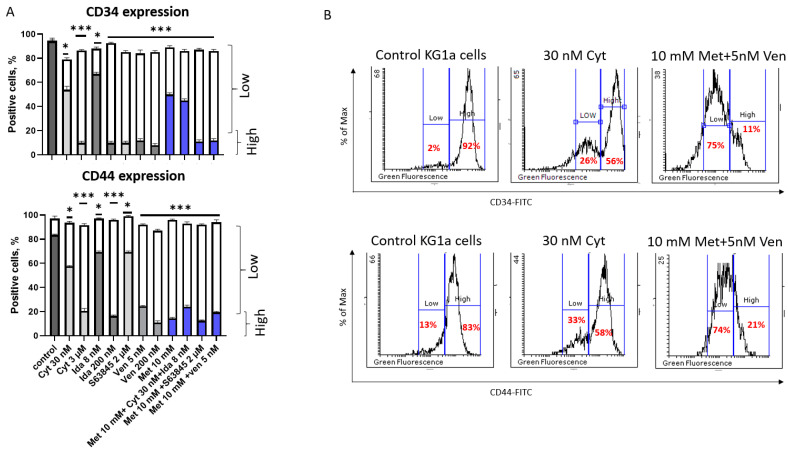
The expression of LSC cell surface markers on KG1a and CD34+ AML cell lines after treatments. (**A**) The expression of LSC markers, CD34 and CD44; (**B**) representative flow cytometry histograms of staining of CD34 and CD44 in unstimulated (control) and stimulated (treated) cells using flow cytometry analysis. The assay was analyzed with 488 nm in the FITC channel. The results are presented as mean ± S.D. Note: * denotes a significant difference between treated vs. control cells with *p* < 0.05, and *** denotes a significant difference with *p* < 0.005. Cyt—cytarabine; Ida—idarubicin; Ven—venetoclax; Met—metformin.

The next step was to evaluate changes in the expression of LSC markers like CD34, CD44, and CD47. We did not find any changes in CD47 expression after different treatments compared with control cells. However, we discovered changes in CD34 and CD44 cell surface protein expression (Figure 5A). After analyzing CD34 and CD44 expression on KG1a cells, we observed that the control cells had mostly 92% CD34^high^ and 83% CD44^high^ populations (Figure 5A,B). However, after different treatments, we also detected CD34^low^ and CD44^low^ populations (Figure 5A,B). We detected CD34^high^ downregulation after treatments with 3 μM of cytarabine, 200 nM of idarubicin, 2 μM of S63845, 5 nM or 200 nM of venetoclax, and a combination of metformin with S63845 or venetoclax (Figure 5A,B). After 30 nM of cytarabine, 8 nM of idarubicin, and 10 mM of metformin treatment alone or in combination, the CD34^high^ population prevailed in KG1a culture (Figure 5A).

Another LSC marker was investigated in KG1a cells after treatments. The percentage of the CD44^high^ population decreased after treatments with almost all the drugs used, except for 30 nM of cytarabine, 8 nM of idarubicin, or 2 μM of S63845 (Figure 5A,B).

### 3.5. Venetoclax, Metformin, and S63845 Alone or in Combination with Conventional Treatment Reduces the Potential of a Tendency to Form Cell Aggregates Due to Changes in the Expression of Markers Involved in Adhesion/Migration/Angiogenesis Processes

**Figure 6:**
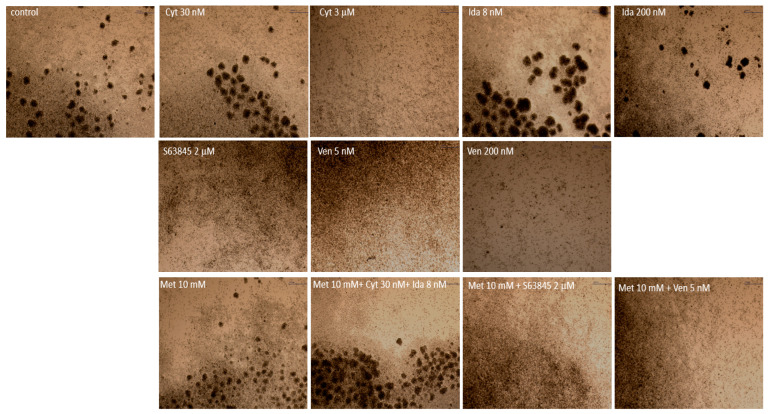
Morphology of CD34+ KG1a cells under light microscopic examination. Original magnification, ×4; Cyt—cytarabine; Ida—idarubicin; Ven—venetoclax; Met—metformin.

It is known that the KG1a cell line has a strong tendency to form cell aggregates due to the expression of different adhesion molecules like integrins, cadherins, and selectins. The expression and activity of these adhesion molecules contribute to the formation of cell aggregates by promoting cell–cell adhesion [18]. We observed that during the culturing of CD34+ KG1a cells, cytarabine (3 μM), 2 μM of S63845, venetoclax (5 nM, 200 nM), and a combination of metformin with S63845 or venetoclax reduced the potential to form cell aggregates (Figure 6). However, lower concentrations of cytarabine (30 nM), idarubicin (8 nM and 200 nM), and 10 mM of metformin alone or in combination with 30 nM of cytarabine and 8 nM of idarubicin did not affect the ability to form cell aggregates in culture (Figure 6).

Here, changes in the expression of markers involved in adhesion/migration/angiogenesis processes such as CD9, CD29, CD31, and CD105 were investigated. Also, the expression of genes like vimentin (*VIM*), neural cell adhesion molecules *NCAM1* and *NCAMII* were analyzed.

**Figure 7:**
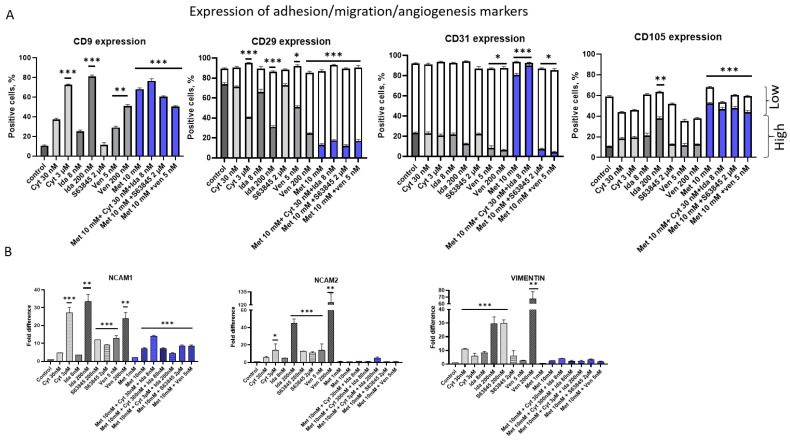
Expression of cell surface markers and genes important for cell adhesion, migration, and angiogenesis on KG1a and CD34+ AML cell lines after treatments. (**A**) The KG1a control and treated cells were labeled with different antibodies to detect cell surface protein expression by flow cytometry; (**B**) *NCAMI*, *NCAMII*, and *VIM* gene expression was detected by qRT-PCR analysis. qPCR results were normalized to GAPDH gene expression, and the relative gene expression was calculated with the ΔΔCt method. The results are presented as mean ± S.D. Note: * denotes a significant difference between treated vs. control cells with *p* < 0.05, ** denotes a significant difference with *p* < 0.01, and *** denotes a significant difference with *p* < 0.005. Cyt—cytarabine; Ida—idarubicin; Ven—venetoclax; Met—metformin.

We detected the upregulation of CD9 after cytarabine, idarubicin, venetoclax, and metformin alone or in combination (Figure 7). However, S63845 did not affect the expression of CD9 on the KG1a cell surface. Also, we did not find significant changes in the expression of CD29, CD31, and CD105 after 2 μM S63845 treatment. However, VIM gene expression after this treatment was upregulated (Figure 7B). Interestingly only metformin alone or in combination with cytarabine and idarubicin upregulated CD31^high^ expression up to 80–90% and also CD105^high^ up to 40–50% (Figure 7A). Metformin in different combinations (Met + Cyt + Ida; Met + S63845; Met + Ven) upregulated the CD9 cell surface marker, and this upregulation correlated with the downregulation of CD29^high^ and the upregulation of CD105^high.^. However, such changes were not accompanied by changes in the expression of the *NCAM1*, *NCAM2* or *VIM* genes (Figure 7B). CD9 was upregulated after treatment with 3 mM of cytarabine (70%), 200 nM of idarubicin (80%), and 200 nM of venetoclax (50%). In this case, CD9 upregulation correlated with the upregulated expression of *NCAM1* or *NCAM2* genes and CD29^high^ cell surface marker downregulation (Figure 7A).

In conclusion, we demonstrated that treatment with cytarabine, idarubicin, venetoclax, metformin, and S63845 in a dose-dependent manner downregulated the strong tendency of CD34+ KG1a cells to form cell aggregates in culture, as well as the expression of leukemic stem cell markers like CD34 and CD44, as well as affected adhesion markers.

## 4. Discussion

The prognosis for patients with acute myeloid leukemia is extremely unfavorable in the long run. According to research, resistance to chemotherapy may be defined by subpopulations of leukemia cells that are more resistant to mitochondrial-mediated apoptosis or have a higher degree of oxidative phosphorylation [7]. Also, these cells are capable of initiating and maintaining the leukemic clonal hierarchy. It is known that AML LSCs require interaction with a niche to maintain their stem cell properties, self-renewal capacities, and resistance to therapy [13]. These interactions are mediated by adhesion molecules, such as integrins and selectins, as well as cell surface receptors and ligands. Also, the niche produces a variety of soluble factors, including cytokines, chemokines, growth factors, and extracellular matrix proteins, which act through signaling pathways, such as the Wnt/β-catenin pathway and the Notch pathway, to maintain LSC stemness.

A permanent cure for AML requires the elimination of LSCs. One way to achieve this is to reduce their ability to interact with the niche and eliminate quiescent AML LSCs. Another way is to develop new strategies for targeting mitochondria-specific oxidative phosphorylation that can hold great therapeutic promise in improving AML treatment efficacy, reducing side effects, and reducing the risk of disease relapse.

In this study, we investigated the anti-leukemic effects of metformin, venetoclax, and S63845 alone or in conjunction with first-line therapy (cytarabine and idarubicin) in the AML leukemic stem-like cell line, KG1a. Targeted drugs such as oxidative phosphorylation inhibitors like metformin (approved for diabetes) and epigenetic modifiers like venetoclax (BCL2 inhibitor, approved for chronic lymphocytic leukemia (CLL), AML) and S63845 (MCL1 inhibitor, experimental) may be combined with first-line chemotherapeutic drugs (cytarabine, idarubicin) to improve AML treatment efficacy. Metformin and venetoclax have been shown to inhibit cancer cell growth. Despite this, little is known about the mechanisms of action and therapeutic impact of the tested medicines in comparison to traditional chemotherapeutics.

**Figure 8:**
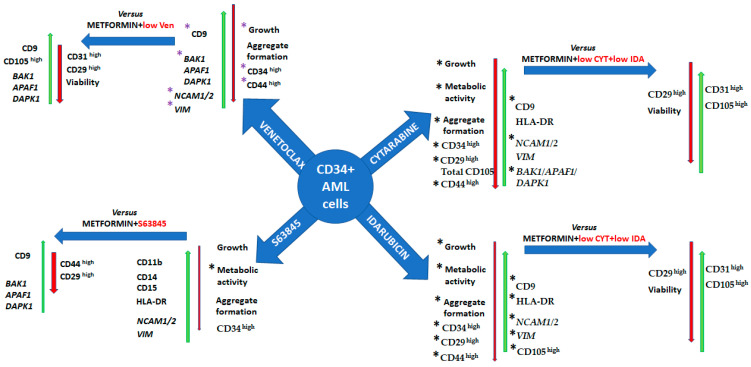
A schematic depiction of chemotherapy’s effect on CD34+ KG1a cells using first-line agents like cytarabine, idarubicin, venetoclax, and S63845 alone vs. in combination with metformin. * denotes a dose-dependent effect. Cyt—cytarabine; Ida—idarubicin; Ven—venetoclax; Met—metformin.

MCL-1 and BCL-2 are typically overexpressed in acute myeloid leukemia and are required for the survival of AML cells and stem cells. In this study, we demonstrate that targeting BCL-2 with venetoclax and/or MCL-1 with S63845 is highly effective for reducing AML cells in culture, which is consistent with recent reports [11,21,22]. We found that the growth and viability of primitive and undifferentiated CD34+ KG1a cells were mostly affected after treatment with venetoclax alone or in combination with metformin. However, the effectiveness of a venetoclax-based low-intensity regimen for R/R AML was disappointing, with a response rate of 21% and a median survival of 3.0 months [4,23]. We determined that a combination of metformin with a lower dose (5 nM) of venetoclax can induce the growth inhibition and metabolic activity of CD34+ chemoresistant AML cells, and these effects can be induced with higher doses of venetoclax as well. We detected that a combination of metformin with venetoclax can boost pro-apoptotic gene expression more than venetoclax alone (Figure 8). Cell cycle analysis revealed an increase in the number of cells in the G0/G1 cycle phase after treatment with venetoclax, which coincides with growth inhibition and apoptosis activation, also correlating with given trends mentioned in the relevant literature [24]. However, a combination of venetoclax with metformin induces the accumulation of cells in the G2/M cell cycle phase. The mechanism by which metformin affects the G2/M cell cycle phase is not fully understood, but several hypotheses have been proposed, like AMP-activated protein kinase (AMPK) activation, mTOR inhibition, the activation of p53, or the alteration of mitochondrial function. It was shown that metformin activates AMPK and that this can lead to cell cycle arrest at the G1/S and G2/M checkpoints by inhibiting the activity of cyclin-dependent kinases (CDKs) and by modulating the expression of cell cycle regulatory proteins [25]. Also, the inhibition of mTOR by metformin may lead to cell cycle arrest at the G2/M phase by interfering with the translation of proteins required for cell cycle progression [25]. Treatments with cytarabine, idarubicin, venetoclax, and a combination of metformin with S63845 increased the expression of pro-apoptotic genes such as *BAK1*, *BAX*, *DAPK1*, and *APAF1*. If we compare the effects of high doses of cytarabine or idarubicin vs. a combination of metformin with a low dose of cytarabine and a low dose of idarubicin, we can find that the combination better reduced viability and also changed cell surface markers (upregulated CD105^high^, CD31^high^ or downregulated CD29^high^) (Figure 8).

The anti-leukemic effect of S63845 (experimental agent) was investigated in this work. S63845 is a small molecule that specifically binds with high affinity to the BH3-binding groove of MCL1. Apart from its mode of action in MCL1-dependent leukemia, little is known about this new inhibitor, S63845, which was described by Kotschy et al. [26]. Although MCL1-dependent leukemias are uncommon, inhibiting one or more of the key pro-survival proteins is critical for fighting AML. In this study, we determined that a 2 μM S63845 treatment of chemotherapy-resistant KG1a cells induced cell growth inhibition and metabolic activity inhibition, and it also induced the accumulation of cells in the S cell cycle phase. Also, we found that S63845 induced myeloid differentiation markers’ (like CD11b and CD14) upregulation up to 30–40%. The combination of S63845 with metformin enhanced the upregulation of pro-apoptotic genes, and CD9, CD105^high^ cell surface markers, also downregulated CD44^high^ and CD29^high^ (Figure 8).

It is known that the KG1a cell line has a strong tendency to form cell aggregates due to the expression of different adhesion molecules like integrins, cadherins, and selectins. The expression and activity of these adhesion molecules contribute to the formation of cell aggregates by promoting cell–cell adhesion [18]. In this study, we evaluated how different anti-proliferative agents can influence the expression of different cell surface markers. First, we characterized KG1a cells. We found that KG1a cells were positive for leukemic stem cell markers like CD34, CD44, and CD47. However, KG1a cells were negative for LSC markers like CD117. It is known that LSC markers like CD34, CD44, CD47, CD38, CD123, CD90 and CD336 play a crucial role in the initiation and perpetuation of the disease. We can conclude that KG1a cells exhibit some properties associated with LSCs that influence the ability to be resistant to differentiation and chemotherapy treatment, but they are not equivalent to primary patient-derived LSCs. Also, in this study, we revealed that KG1a cells were negative for differentiated hematopoietic cell markers like CD3, CD4, CD8, CD11b, CD14, and CD19. However, we found that cells were positive for CD5, CD7, CD10, CD13, CD29, CD31, CD33, CD36, CD45, CD105, CD166, and HLA-ABC and were negative for CD73, CD90, and HLA-DR. Our findings partly correlated with previous studies. However, we did not find many studies on cell surface markers in CD34+ chemotherapy-resistant KG1a cells [27].

Next, we determined that the anti-proliferative effect of cytarabine, idarubicin, venetoclax, metformin, and S63845 were correlated with the reduction in CD34^high^ and CD44^high^ but not with CD47 cell surface markers. It was shown in the literature that cancer cells expressing higher levels of CD44 are more resistant to apoptosis [13,28]. It is known that low human leukocyte antigen (HLA)-DR expression might compromise CD4+ T-cell-mediated anti-tumor immunity [29]. Here, we showed that HLA-DR was upregulated after treatments, and this could enhance their recognition by T cells, leading to the activation of anti-leukemia immune responses. This can potentially facilitate the clearance of leukemia cells by the immune system and contribute to improved treatment outcomes.

Also, we detected changes in the expression of markers such as CD9, CD29, CD31, and CD105 involved in adhesion/migration/angiogenesis processes. We detected the upregulation of the CD9 cell surface marker in a dose-dependent manner after treatment with cytarabine, idarubicin, and venetoclax. Metformin alone or in combination with other treatments studied also upregulated CD9 expression. We can see that metformin can boost the effects of S63845 and venetoclax in the upregulation of the CD9 cell surface marker in KG1a cells (Figure 8). However, only metformin alone or in combination with cytarabine and idarubicin upregulated CD31^high^ expression. This finding correlated with results indicating that metformin did not induce pro-apoptotic gene expression in CD34+ KG1a cells. The upregulation of CD31 in AML cells may be associated with therapeutic resistance mechanisms. CD31-mediated signaling pathways may contribute to the activation of pro-survival and anti-apoptotic pathways.

Adhesion molecules play a very important role in the communication process between AML cells and the bone marrow niche. Also, in this study, we investigated changes in neural cell adhesion molecules 1 and 2, as well as vimentin genes. We found the upregulation of these genes after treatment with higher doses of cytarabine, idarubicin, and venetoclax. Metformin alone or in combination with other treatments did not upregulate *NCAM2* and *VIM* but slightly upregulated the *NCAM1* gene. It is known that NCAM 1/2 are members of the immunoglobulin superfamily of cell adhesion molecules. NCAM1 expressed on AML cells may participate in interactions with endothelial cells lining blood vessels within the bone marrow microenvironment. These interactions are crucial for the extravasation of leukemia cells from the bloodstream into the bone marrow, a process essential for leukemia homing and progression. Vimentin is a hallmark marker of epithelial-to-mesenchymal transition (EMT), a cellular process associated with increased cell motility, invasiveness, and meta-static potential in cancer. In AML, vimentin upregulation may be associated with EMT-like phenotypic changes that enhance cell migration, invasion, and resistance to apoptosis.

The results of this study suggest that combining metformin with other therapies like cytarabine, idarubicin, venetoclax, or S63845 may allow for dose reductions in individual agents, which can help mitigate treatment-related toxicities while maintaining or even enhancing efficacy. This is particularly relevant in resistant AML treatment, where chemotherapy-induced toxicities can be dose-limiting factors. The results of our study may lead to the inclusion of metformin as adjuvant therapy in the first-line treatment protocols of patients who cannot receive aggressive chemotherapy, thus facilitating the development of individualized treatment strategies. It is known that metformin acts by targeting metabolic pathways and potentially affecting leukemic stem cells. This may help reduce the risk of disease relapse or progression in patients with AML. The long-term use of metformin as a part of maintenance therapy may also contribute to durable remissions and improved long-term outcomes. Of course, close monitoring and individualized treatment approaches may be necessary to optimize the use of metformin in this setting and minimize the risk of adverse events such as vitamin B12 deficiency, megaloblastic anemia, and thrombocytopenia.

In conclusion, we believe that metformin has the potential to be used as an enhancer in the treatment of resistant-to-first-line-chemotherapy AML cells. Also, we believe that the results of our study will stimulate further research and the potential use of changes in the expression of cell surface markers in the development of new therapeutic strategies or diagnostic/prognostic tools.

## Data Availability

The data presented in this study are available upon request from the corresponding author.

## Supplementary Materials

The following supporting information can be downloaded at: https://www.mdpi.com/article/10.3390/genes15050648/s1, Supplement S1: Metformin as an Enhancer for the Treatment of Chemoresistant CD34+ Acute Myeloid Leukemia Cells.
