# Supplementary figures and images for: Metformin as an Enhancer for the Treatment of Chemoresistant CD34+ Acute Myeloid Leukemia Cells
Source: Genes (Basel). 2024 May 20;15(5):648. doi: 10.3390/genes15050648 (PMC11121461; doi:10.3390/genes15050648)

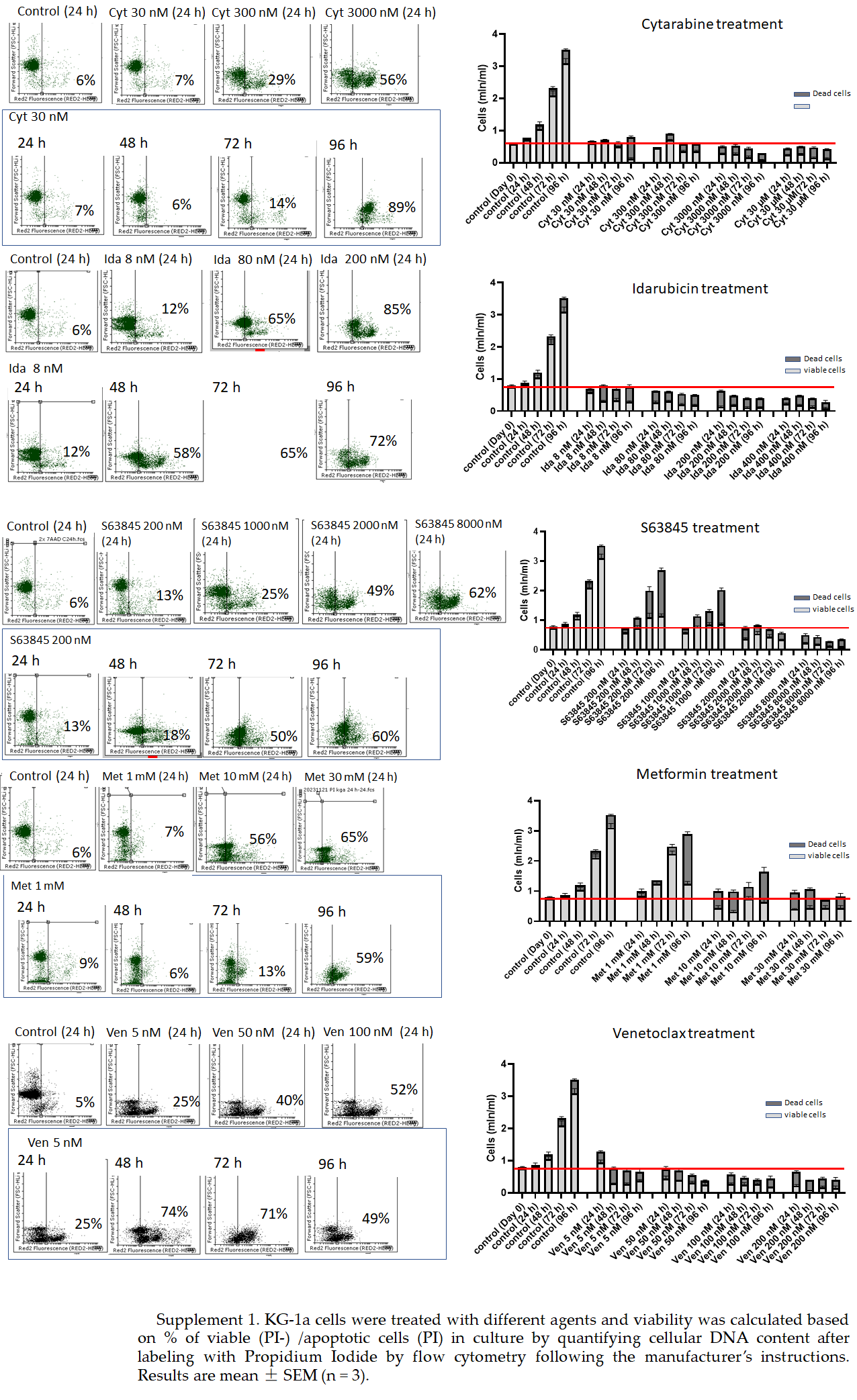

Supplement: Supplementary file 1 [file genes-15-00648-s001.zip › Supplement S1.tif]
